# From Checklists to Tools: Lowering the Barrier to Better Research Reporting

**DOI:** 10.1371/journal.pmed.1001910

**Published:** 2015-11-24

**Authors:** The PLOS Medicine Editors

## Abstract

The *PLOS Medicine* Editors reflect on the future of research reporting checklists.

Writing research articles is no trivial task. As complex technical documents that often mark the culmination of many years of work by many contributors, they can require considerable coordination even to assemble an initial draft. Ensuring accurate and complete reporting is critical to informing subsequent work and, especially in medical research, to the thoughtful interpretation of research findings with potentially profound consequences for clinical research and practice. While waste in research happens at many levels, it would seem that accurately and completely reporting research is one area that should be readily amenable to minimizing wasted effort [1]. Disappointingly, however, research on research indicates that authors and editors are not doing well in this regard [1].

As journal editors, we are interested in efforts to improve reporting in published research, and, together with our colleagues at other journals, have proudly featured the efforts of those researchers who develop research reporting guidelines [2]. It has even been argued that the CONsolidated Standards Of Reporting Trials (CONSORT) [3] and Preferred Reporting Items for Systematic Reviews and Meta-Analyses (PRISMA) [4] reporting guidelines are some of the most important academic works we’ve published [5], and they are certainly highly read and cited. *PLOS Medicine* requires that certain checklists be included on submission for research studies, including CONSORT for clinical trials [3], STROBE for observational studies [6], Standards for Reporting of Diagnostic Accuracy (STARD) for diagnostic accuracy studies [7], and PRISMA for systematic reviews [4], and we encourage the use of other relevant guidelines where they exist. There is evidence that endorsement of CONSORT by journals increases the completeness of reporting for randomized controlled trials even if reporting remains suboptimal [8]. More consistent implementation of checklists by journals and authors should improve reporting further, but could the checklists themselves also evolve to achieve the same ends?

Despite general consensus among editors in favor of checklists, a feeling of saturation may be setting in for some authors. Last month, another important reporting guide extension joined the guidelines already published in *PLOS Medicine*: the REporting of studies Conducted using Observational Routinely-collected Data (RECORD) statement, an extension to STROBE for reporting observational studies that use routinely collected health data [9]. Peer review of the guideline was supportive and constructive, but one reviewer took the opportunity to express exasperation about the proliferation of reporting guidelines in general: “How many more unenforceable proclamations and checklists do we need?”

While the reviewer also noted support for the authors’ efforts in developing the guideline, this frustration may be familiar to many. For some prospective authors, journal requirements for providing a relevant checklist can feel like yet another hurdle along the journey to publication. What’s more, for those authors who are keen to use a guideline to help develop their work, identifying which reporting guidelines are available and relevant can be a substantial task. As we write, there are 284 reporting guidelines listed on the Enhancing the QUAlity and Transparency Of health Research (EQUATOR) network’s website [10], and for some reporting guidelines, there are many extensions. For example, there are ten official extensions to the CONSORT reporting guidelines [11].

If there is value in reporting guidelines, as we believe there is, how can the barrier to use be reduced so that the outputs of reporting guideline development are not seen as “unenforceable proclamations and checklists”? Education and training is likely to be one component [12], but substantial inroads might also be made if reporting checklists became integrated within authoring tools [13]. In this area, some interesting work is beginning to be done.

In a recently published study in *BMC Medicine*, Isabelle Boutron and colleagues have tested a writing aid tool that brings the application of the reporting guidelines to the heart of the writing process [14]. The tool, CONSORT-based WEB tool (COBWEB), is used while authors write the methods sections of their clinical trial. By guiding the authors through a series of questions based on CONSORT and the CONSORT extension for nonpharmacologic treatments and generating a formatted Word document, the tool ensures that a paper’s first draft includes many of the key requirements for reporting trials. Perhaps unsurprisingly, when tested in a randomized trial of 41 students tasked with writing the methods section of trials based on real trial protocols, those who used the tool were able to follow the tool’s instructions, and their methods sections were more completely reported than those in the control group. The tool has already attracted enthusiastic support and drawn comparisons to the Review Manager (RevMan) tool and extensions that already help researchers working on Cochrane systematic reviews prepare the text of their review [13]. Would COBWEB improve reporting by a group of experienced researchers writing up a new trial, can it be broadened for use beyond the methods sections and beyond trials, and will there be wide enough uptake for the tool to have an impact? Further development will likely be needed before the tool will realize its potential, but the concept of moving from postwriting checklists to authoring tools is an intriguing one that has the potential to be a helping hand rather than a postwriting chore.

Elsewhere, the EQUATOR network is working with the start-up company Penelope [15] to develop a web tool that aims to help authors identify relevant reporting guidelines more intuitively [16]. Perhaps more interesting is Penelope’s main product under development [17], which checks a manuscript automatically for predictable errors and missing information; this includes highlighting potentially relevant checklists but goes further by identifying other commonly missed or incompletely reported pieces of information that are required for publication of a research article, such as citations, tables, and ethics statements, and by even scrutinizing *p*-values [15]. The target customers are publishers [15], which would mean that the software would not be applied until after a research manuscript has been finalized for submission. If software products that can recognize what has been written (and therefore what is missing) turn out to be useful, time-saving tools, it may be that institutions and individual authors will see the value in applying this type of software earlier in the writing process too. Ideally, evidence-based community priorities for essential items in reporting will eventually be integrated at study design.

Could there be a knock-on advantage of integrating items from reporting checklists into authoring tools? If we allow ourselves to dream of the article of the future, we may not need checklists to be submitted as supporting files that refer to locations within a pdf or html version of a final published article. Perhaps, the locations of reporting items generated by authoring tools could be encoded into machine-readable metadata that follow the manuscript through to publication; this would give interesting options for displaying content, but more importantly, by providing rich datasets for research on research reporting, it would facilitate studies on how well reporting guidelines are achieving their aims. Of course, such an effort would require substantial collaboration across publishers and platforms. In the meantime, completely and accurately reported research studies, even without further bells and whistles, remain a highly worthwhile goal.

So, how many more unenforceable proclamations and checklists do we need? The answer might be that it doesn't matter how many are generated if reporting guidelines can evolve into genuinely useful and intelligent author aides that become as ubiquitous as citation software.

## Abbreviations

COBWEB: CONSORT-based WEB tool
CONSORT: CONsolidated Standards Of Reporting Trials
EQUATOR: Enhancing the QUAlity and Transparency Of health Research
PRISMA: Preferred Reporting Items for Systematic Reviews and Meta-Analyses
RECORD: REporting of studies Conducted using Observational Routinely-collected Data
RevMan: Review Manager
STARD: Standards for Reporting of Diagnostic Accuracy
